# Tear of peroneus longus in long distance runners due to enlarged peroneal tubercle

**DOI:** 10.1186/2052-1847-6-1

**Published:** 2014-01-13

**Authors:** Ezequiel Palmanovich, Lior Laver, Yaron S Brin, Iftach Hetsroni, Meir Nyska

**Affiliations:** 1 Orthopedics department – Meir Medical Hospital, Sacker School of Medicine Tel Aviv University, Kfar – Saba, Israel

**Keywords:** Enlarged peroneal tubercle, Peroneus tendon tendon tear, Runners lateral ankle pain, Surgical treatment

## Abstract

**Background:**

Tear of the Peroneus longus in association with a prominent peroneal tubercle is rare.

**Case presentation:**

Recently we treated two long distance runners who developed lateral ankle pain. Maximum tenderness was located over the lateral surface of the heel in the area of the peroneal tendons. Imaging disclosed a tear of the peroneus longus at the area of the peroneal tubercle.

**Conclusion:**

Following resection of the peroneal tubercle and repair of the peroneus longus, both patients regained full activity with no pain. This report describes the clinical presentation and surgical management of this rare injury.

## Background

The close relation between the peroneal tendon and the peroneal tubercle has been well described by Sarrafian [1]. Other studies also described enlargement of the peroneal tubercle [2] leading in some cases to stenosing tenosinovitis [3-5].

Tendinopathies around the ankle are commonly described in long distance runners [6-8]. However, to the best of our knowledge, there is no description of peroneus Longus tendinopaty and tear due to a prominent peroneal tubercle.

In this report, we describe two cases of long distance runners who suffered a tear of the peroneus longus associated with an enlarged peroneal tubercle. We discuss the clinical presentation and surgical management.

## Case presentation

### Case 1

A 58-year-old marathon runner presented with pain in the lateral aspect of the ankle during running. Pain was severe, with peak severity after 30 km of running and forcing him to stop training.

Localized tenderness over the lateral aspect of the foot was present on physical examination. It worsened with inversion of the ankle due to stretching of the peroneal tendons. Swelling over the lateral aspect of the calcaneous was noted, with no rubor.

**Figure 1:**
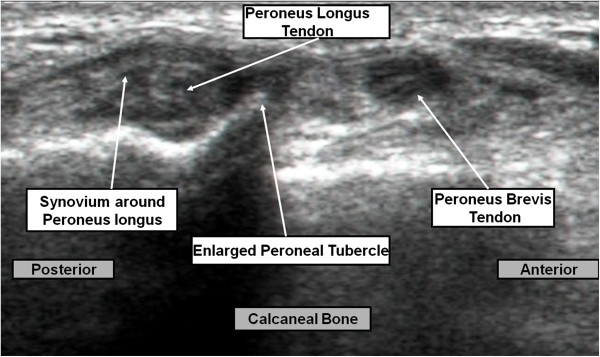
Ultrasound showing hypertrophic peroneal tubercle and the thickened peroneus longus with synovitis around the tendon.

**Figure 2:**
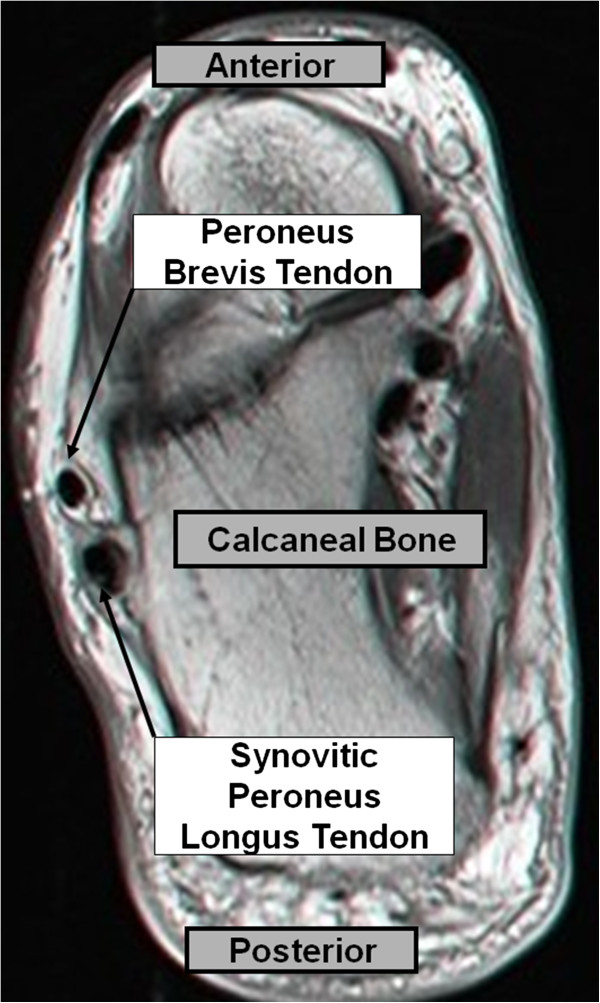
Axial MRI view showing the synovitic thickened peroneus longus.

Ultrasound (US) at the area of maximum tenderness revealed a hypertrophic peroneal tubercle and a thickened peroneus longus with synovitis around the tendon (Figure 1). Magnetic resonance imaging (MRI) showed a synovitic, thickened peroneus longus tendon (Figure 2).

**Figure 3:**
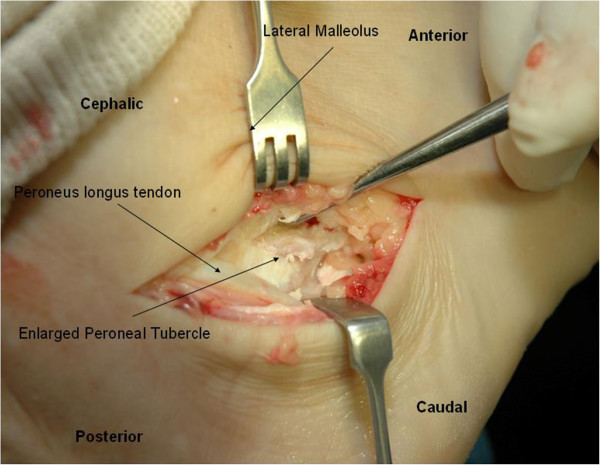
Lateral approach to the lateral side of the ankle - tear of the peroneus longus just over the enlarged peroneal tubercle.

Nonoperative management consisting of rest and physical therapy did not provide relief. The patient underwent excision of the hypertrophic peroneal tubercle and repair of the peroneus longus tendon that had a partial longitudinal tear (Figure 3).

Post-operative management included immediate walking with full weight bearing. Physiotherapy was initiated 3 weeks after surgery and he returned to running activity at 6 weeks. Four months after surgery, he finished the New York marathon.

### Case 2

A sixty-year-old male jogger with no history of trauma developed gradual pain on the lateral aspect of the foot. He stopped jogging as a result.

Upon physical examination, localized tenderness was found over the lateral aspect of the foot. It worsened with inversion that generating peroneal stretching and with palpation over the groove of the peroneus longus tendon.

**Figure 4:**
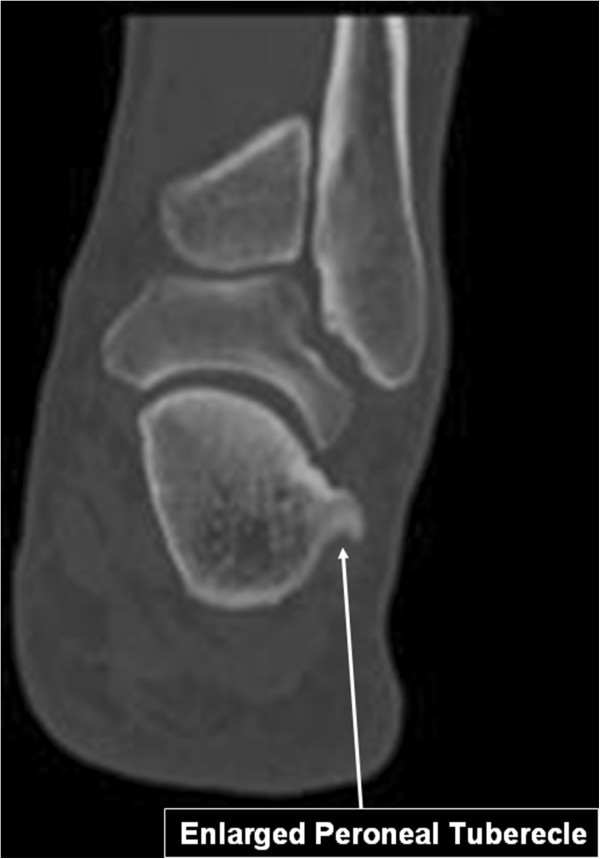
Coronal CT scan view showing the enlarged peroneal tubercle.

**Figure 5:**
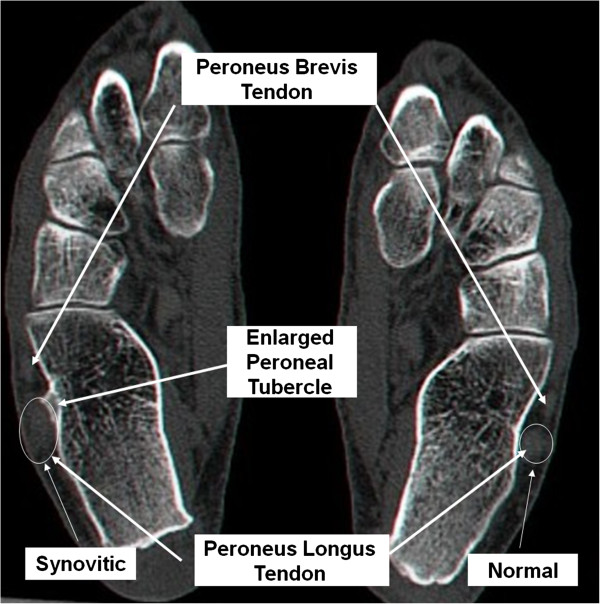
Axial CT scan view showing the enlarged peroneal tubercle and thickened peroneus longus.

Computerized Tomography (CT) revealed a hypertrophic peroneal tubercle (Figure 4) and in sagital plane saw the enlarged peroneal tubercle and the thickened peroneus longus compared to the other healthy calcaneous (Figure 5).

Conservative treatment resulted in no improvement. Surgical exposure of the lateral ankle disclosed a partial longitudinal tear of the peroneus longus over the enlarged tubercle. Excision of the tubercle was performed followed by suture of the peroneus longus. We noted an anomaly of the peroneus brevis, which was inserted to the peroneal tubercle.

Postoperative management included immediate full weight bearing and initiation of physiotherapy 3 weeks after surgery. He returned to jogging only a few weeks later.

## Conclusion

The true incidence of peroneal tendon tears is unknown, with estimates ranging from 11% to 37% in cadaver dissections and up to 30% in patients undergoing surgery for ankle instability [9]. Hyer et al. studied 114 calcanei, in which the peroneal tubercle was present in 90.4% [10].

Most if not all publications, report a stenosing tenosinovitis without a tear of the peroneal tendon. We did not find any reports of a non-traumatic tear of the peroneus longus due to a hypertrophic peroneal tubercle. In 2006, Sugimoto reported three cases of peroneal tenosynovitis due to an enlarged peroneal tubercle in association with Sural nerve entrapment [11].

In the 2 cases reported here, the gradually increasing lateral ankle pain and decrease performance of the runners led to suspected chronic injury of the peroneal tendons. Exploration with simple radiography or CT can demonstrate the peroneal tubercle and provide information about existing hypertrophy. Sobel et al. stated that plain radiography using the Harris Heel view could be used to demonstrate the presence of an enlarged peroneal tubercle [5]. In addition, MRI can show peroneal tendon damage and bone marrow edema.

In our patients, accurate diagnosis was made by US, CT and MRI. US dynamically demonstrated the friction of the tendon over the enlarged peroneal tubercle. This is the preferred method for demonstrating the pathological mechanism.

We think that the tear in our patients was caused by chronic friction of the peroneus longus tendon over the hypertrophic peroneal tubercle, leading to longitudinal tear. The tear was located exactly over the enlarged peroneal tubercle.

Operative treatment consisting of excision of the tubercle and suture of the tendon, leading to good results with rapid return to sport activity. Additional studies are needed to enable better understand of the physiopathology of the hypertrophic peroneal tubercle and the kinematics of the peroneal tendon after tubercle resection.

Chronic friction of the peroneus longus tendon over a hypertrophic peroneal tubercle can lead to a longitudinal tear. Simple excision of the tubercle with suturing of the tendon yielded good results.

## Consent

Written informed consent was obtained from the patient for publication of this Case report and any accompanying images. A copy of the written consent is available for review by the Editor of this journal.

## Competing interests

The authors declare that they have no competing interests.

## Authors’ contributions

Made substantial contributions to conception and design, or acquisition of data, or analysis and interpretation of data: EP, LL, YB. Involved in drafting the manuscript or revising it critically for important intellectual content. EP, IH, MN. Final approval of the version to be published, EP, IH, MN. All authors read and approved the final manuscript.

## Pre-publication history

The pre-publication history for this paper can be accessed here:

http://www.biomedcentral.com/2052-1847/6/1/prepub

## References

[B1] Sarrafian SK Sarrafian SK Osteology Anatomy of the Foot and Ankle Location, Publisher, pages, year 1993 2 Philadelphia, Lippincott: Anatomy of the foot and ankle 63 64

[B2] Pierson JL Inglis AE Stenosing tenosynovitis of the peroneous longus tendon associated with hypertrophy of peroneal tubercle and os perineum. A case report J Bone Joint Surg Am 1992 74 440 442 1548274

[B3] Bruce WD Christofersen MR Phillips DL Stenosing tenosynovitis and impingement of the peroneal tendons associated with hypertrophy of the peroneal tubercle Foot Ankle Int 1999 20 464 467 10.1177/107110079902000713 10437932

[B4] Chen YJ Hsu RW Huang TJ Hypertrophic peroneal tubercle with stenosing tenosynovitis: the results of surgical treatment Changgeng Yi Xue Za Zhi 1998 21 442 446 10074731

[B5] Sobel M Pavlov H Geppert MJ Thompson FM DiCarlo EF Davis WH Painful os peroneum syndrome: a spectrum of condition responsible for plantar lateral foot pain Foot Ankle Int 1994 15 112 124 10.1177/107110079401500306 7951939

[B6] Fallon KE Musculoskeletal injuries in the ultramarathon: the 1990 Westfield Sydney to Melbourne run Br J Sports Med 1996 30 319 323 10.1136/bjsm.30.4.319 9015594 PMC1332416

[B7] Bishop GW Fallon KE Musculoskeletal injuries in a six-day track race: ultramarathoner’s ankle Clin J Sport Med 1999 9 216 220 10.1097/00042752-199910000-00006 10593216

[B8] Hutson MA Medical implications of ultra marathon running: observations on a six day track race Br J Sport Med 1984 18 44 45 10.1136/bjsm.18.1.44 PMC1858850 6722427

[B9] Squires N Myerson M Gamba C Surgical treatment of peroneal tendon tears Foot and Ankle Clin N Am 2007 12 675 695 10.1016/j.fcl.2007.08.002 17996622

[B10] Hyer C Dawson J Philbin T Berlet G Lee T The peroneal tubercle: description, classification, and relevance to peroneus longus tendon pathology Foot Ankle Int 2005 26 947 950 16309609 10.1177/107110070502601109

[B11] Sugimoto K Takakura Y Okahashi K Tanaka Y Ohshima M Kasanami R Enlarged peroneal tubercle with peroneus longus tenosinovitis J Orthop Sci 2009 14 330 335 10.1007/s00776-008-1326-3 19499301

